# Knowledge, opinions and experiences of researchers regarding ethical regulation of biomedical research in Benin: a cross-sectional study

**DOI:** 10.1186/s12910-022-00857-x

**Published:** 2022-11-19

**Authors:** Flore Gangbo, Grâce Quenum, Fernand Aimé Guédou, Martial Boko

**Affiliations:** 1 Department of Human Biology, Faculty of Health Sciences, University of Abomey-Calavi, Cotonou, Benin; 2 Ophthalmology Clinic at the National Teaching Hospital Hubert Koutoukou Maga, Avenue Jean-Paul II, 01BP 386, Cotonou, Benin; 3 Laboratory of Sexually Transmitted Infections, Cotonou, Benin; 4 National Ethics Committee for Health Research, Ministry of Health, Cotonou, Benin; 5 Court of Appeal, Ministry of Justice and Human Rights, Cotonou, Benin

**Keywords:** Ethical regulation, Biomedical research, Knowledge, Experiences, Researchers, Benin

## Abstract

**Background:**

Ethics in biomedical research is still a fairly new concept in Africa. This work aims to assess the knowledge, attitude and experiences of Beninese researchers with regard to the national ethical regulatory framework of biomedical research in Benin.

**Methods:**

This was a cross-sectional and descriptive study, involving all the researchers fulfilling the inclusion criteria. Data were collected through a face-to-face interview using a questionnaire and analysed. Proportions and means were calculated with their confidence intervals and standard deviations, respectively.

**Results:**

Of the 110 participants included in the study, 40.9% were medical lecturers and 71.1% had been involved in more than 10 biomedical research as researcher. Less than three quarters (69.1%) were able to correctly quote the basic principles from Belmont report. The quarter (25.45%) of them knew the attributions of the National Ethics Committee for Health Research (CNERS in French) and 38.2%, the content of the legislation on health research ethics in Benin. The common ethical rules were known by 69.1% of the participants. A quarter (25.5%) of participants said they always present the study’s briefing note to their study participants and 62.7% said they systematically request informed consent. For those who do not present the briefing note to participants, the main reasons provided were the researchers’ difficulties in writing the note in plain language and the participants’ limitation in understanding it.

**Conclusions:**

The foundations of a good ethical framework for health research exist in Benin. However, the deployment and use of the various legal texts deserve to be improved.

## Background

Although not being the only continent with poor ethical compliance in health research, Africa seems more conducive to it by the lack of regulatory structures or their very limited actions when they do exist [1]. In a study conducted on research misconduct among Nigerian researchers, half of the respondents (50.4%) were aware of a colleague who had committed misconduct, defined as “non-adherence to rules, regulations, guidelines, and commonly accepted professional codes or norms” [2]. Indeed, ethics in biomedical research is still a fairly new concept in Africa and little understood by research stakeholders, including researchers. In a study designed to develop a module for online training in research ethics based on the Nigerian National Code of Health Research Ethics, and involving biomedical researchers, the mean pre-test score was 53.9 (SD 26.4) [3].

Ethics in research involving the human person is articulated around three principles: respect for the human person, beneficence and justice, which derive from human rights. Each of these principles is broken down into multiple rules to which researchers are subject. The most common consensuses are summarised into the guidelines of the Council for International Organizations of Medical Sciences [4].

The effective protection and safety of research participants certainly depends on the good knowledge and appropriation of regulation texts by researchers and their commitment to comply with them, in person or by delegation, which place them at the heart of this protection.

In addition, close collaboration between the researcher and the regulatory bodies, including particularly the Research Ethics Committee (REC), is of upmost interest. This requires from the researchers a good knowledge of the regulatory mechanisms and texts and the institutions that manage them.

The design and implementation of ethically and scientifically valid research in any country should be guided by a set of rules and regulations based on global ethical principles but domesticated within local laws, regulations and culture [3]. In Benin, the law N° 2010–40 of December 8, 2010 related to the code of ethics and deontology for health research in the country, sets the rules for the legal framework of health research. To date, four committees have been created to ensure the regulation of health research. These include the National Ethics Committee for Health Research (CNERS in French) set up in 2009 and three institutional ethics committees, namely the Research Ethics Committee of the Institute of Applied Biomedical Sciences, the Local Ethics Committee for Biomedical Research of the University of Parakou and the Ethics Committee for Research in Health Sciences, created in 2019, 2015 and 2017 respectively.

As ethical approval has become mandatory for funding research and publishing its findings, researchers increasingly submit their research for ethical approval prior to their implementation. However, little is known about the extent to which they actually comply with ethical principles (beyond study protocol submission) and even about their level of knowledge, their perspectives and experiences related to the national ethical regulation mechanism and texts. Such information is important to infer the researchers needs for support, for a better compliance with ethical rules with the perspective of an effective protection of research participants.

The aim of this work was thus to assess the knowledge, attitudes and perceptions of biomedical researchers, regarding the ethical regulation of biomedical research in Benin.

Specific objectives were to:

- Evaluate the awareness of the researchers regarding the ethical regulation of biomedical research in Benin and specifically the national ethics committee for health research (existence, functioning and ascriptions).
- Analyse their opinions and experiences related to the ethical guidelines governing medical research.

## Setting, methods and materials

### Setting

This study targeted key institutions conducting health research in the economical capital city of Cotonou and in two main surrounding cities which were Porto-Novo and Abomey-Calavi.

These institutions included:

- Five (05) university hospitals, namely the National university Hospital (Hubert Koutoukou Maga), the Mother and Child Hospital (CHUMEL), the regional hospital of Oueme-Plateau (CHUD-OP), the District hospitals of Suru-Léré and Abomey-Calavi / Sô-Ava;
- Eight (08) institutes conducting health related research, including the Faculty of Health Sciences; the Entomology Research Center (CREC); the Center for Research in Human Reproduction and Demography (CERRHUD); the National Malaria Programme (PNLP) the Institute of Applied Biomedical Sciences (ISBA); the Institute for Research and Development (IRD); the Beninese Association for Social Marketing and Health Communication (ABMS/PSI) and Plan International Benin.

## Method and materials

### Study design and sampling

It was a cross-sectional and descriptive study, which included researchers involved or likely to be involved in biomedical research. These were those who have participated as investigator or co-investigator in at least one biomedical research within the last three years; were affiliated with one of the aforementioned research institutions; and finally provided their informed consent to participate in the study. It was an exhaustive sampling as all the researchers that met the above-mentioned criteria were recruited for the study. However, only the researchers that answered at least half of the study questionnaire were included in the analysis, as the exclusion criterion was the uncompletedness of the questionnaire (more than half of the questions left without answers).

### Data collection, processing and analysis

Data were collected using a questionnaire administered in face-to-face interview or self-administered (physically or online). The variables collected were relative to:

- The socio-professional profile of the participants (professional background, research activities and responsibilities assumed over the past three years);
- The knowledge of participants in connection with the national ethics committee for health research (name, year of creation, attributions and operation) and the various regulatory texts and fundamental ethical principles governing biomedical research in Benin;
- The experiences and opinions of researchers in terms of respecting ethical rules during research (obtaining ethical approval, informed consent process, monitoring and reporting of adverse events to the ethics committee).

Collected data were entered, cleaned and analysed using the software Epidata version 3.5.3 and SPSS version 2.1. All the variables were categorical (either originally or by transformation) and were therefore described by proportions with their 95% confidence intervals. Variables related to knowledge were treated in three modalities (“correct answers”, “incorrect answers” and “no answer or unknown”) while those related to experiences and opinions were presented in a varying number of modalities.

## Results

A total of 231 potential participants were approached, of which 89 were ineligible (78 did not provide their informed consent and 11 had not been involved in any research activities for the last 3 years). Thus, data were collected from 142 participants. But only 110 were included in the analysis as 32 left more than half of the questions without answers and were thus excluded from the analysis. Results are presented as it follows.

### Socio-demographic and professional profile of participants

Among the 110 eligible participants, 86 (77.8%) were men and 24 (22.2%) were women. Medical lecturers and specialists predominated, representing respectively 40.9% (45/110) and 36.4% (40/110) of the sample. The remaining respondents break down as follows: holders of doctoral degrees in medicine (9.1%) or other disciplines (9.1%) or holders of various master’s degrees (4.5%) (Table 1).

**Table 1:** Sociodemographic and professional characteristics of participants (N = 110)

Characteristics	Number	(%)
*Sex*		
Male	86	77.8
Female	24	22.2
Total	110	100.0
*Professional categories*		
Medical lecturers	45	40.9
Specialists physicians	40	36.4
Medical doctors	10	9.1
Holders of other doctoral degrees	10	9.1
Holders of master degrees	05	4.5
Total	110	100.0
*Position held in the last research study*		
Promotor	33	30.0
Investigator	10	9.1
Coordinator	24	21.8
Promotor and coordinator	16	14.5
Investigator and coordinator	02	1.8
Data not available	25	22.7
Total	110	100.0

### Responsibilities assumed by participants in research projects

The positions held by the participants during their last three research studies, included promoters (30.0%), coordinators (21.8%), both promoters and coordinators (14.5%), investigators (9.1%) or both investigators and coordinators (1.8%). It was mainly specialist physicians and medical lecturers who held the position of promoters (Table 1).

### Types of research conducted by participants

Epidemiological and clinical studies were the commonest types of research conducted by participants, as they were cited by 69.1% and 63.6% of participants, respectively. They were followed by biomedical (30%), health system (8.2%) and traditional medicine research (5.5%).

### Knowledge of participants in relation to CNERS and regulatory texts

#### Knowledge related to CNERS

While all the respondents knew of the existence of the committee and its acronym, “CNERS”, 57.3% of them were able to correctly define the latter. More than two thirds (68.2%) of the participants abstained from answering the question related to the year of creation of CNERS. Of the 35 participants who responded, 42.9% (n = 15) gave the correct answer (which is 2007). For 57.1% (n = 20), the answers were incorrect: indeed, 16 (45.7%) mentioned the year 2010 (which is instead the year of the promulgation of the Law N° 2010-40 of December 8, 2010 on the ethics and deontology code for health research in Benin) and 4 (11.4%) indicated the year 2013 (which is rather that of the issuance of the Decree No. 2013-48 of February 11, 2013 related to the composition, attributions and functioning of CNERS).

Regarding CNERS attributions, 25.5% of the participants provided the correct answers, 68.2% the wrong ones while the remaining 6.3% declared that they did not know these responsibilities. The procedure for appointing permanent and non-permanent members was known to less than half of the participants i.e., 40.0% and 35.5% respectively.

Most of the respondents (82.7%) were able to correctly describe the entire process leading to the obtention of the ethical approval of a study protocol from CNERS. However, 17.3% of the participants (wrongly) thought that the ethical approval is issued immediately after completion of the protocol examination session by CNERS.

#### Knowledge related to the fundamental principles and texts of the biomedical research ethics in Benin

The participant’s knowledge was evaluated about the basic ethics principles of the Belmont report and the fundamental texts guiding human research ethics in Benin.

#### Number and types of basic ethics principles

**Fig. 1:**
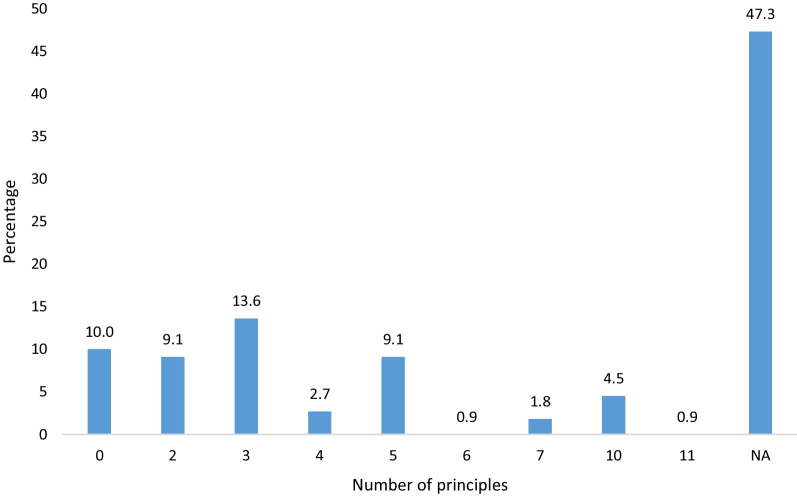
Distribution of participants according to the number of cited ethics principles (from Belmont report) (N = 110). *NA* No answers

About one in 10 researchers were able to indicate the exact number (which is three) of the basic ethics principles guiding the supervision of biomedical research in Benin. Almost half of participants abstained from completing this item (Fig. 1). Respect for the human person was by far the most mentioned principle (45.5%). On the other hand, those of beneficence and justice were cited by 27.3% (n = 30) and 14.5% of respondents (n = 16) respectively.

#### Qualifications required to be an investigator

For respectively 58 (52.7%) and 53 (48.2%) participants, being a specialist or a general practitioner were the minimal qualifications required to be an investigator in biomedical research. Other qualifications such as having paramedical (37.3%) or a master’s (35.5%) degrees were suggested. For 14.5% of participants (n = 16), no qualification was required.

#### Legal responsibility of researchers, protection of research participants and existence of a directory of research work

The proportions of participants who were aware of some key items of the national ethics code for biomedical research were as follows:

- Legal responsibility of researchers: 34.5% of participants.
- Compensation schemes for research participants: 40%.
- Concept of vulnerable person: 33.6%.
- Legal criteria for the assessment of the benefits and risks: 20%.
- Legal obligation to take out insurance for research: 28.2%.
- Conditions for inclusion of vulnerable people: 25.5%.

Finally, 47.3% of participants were aware of the existence of a national directory of biomedical research.

#### Ethical governance regarding clinical research and research on traditional medicine

Some questions focused on the ethical aspect of drug development and marketing including traditional medicine. A little less than half (47.3%) of the participants agreed on the existence of a legal regulation concerning research on traditional medicine. The majority of participants agreed on the existence of legal regulations concerning drugs, their marketing and the distribution of medical devices (Table 2).

**Table 2:** Distribution of participants according to their knowledge of the existence of drug and other health products regulation (N = 110)

Items	Number (%)		
	Yes	No	Unknown
Existence of rules on drug registration and marketing	97 (88.2)	1 (0.9)	12 (10.9)
Existence of regulation on marketing and distribution of medical devices	85 (77.3)	2 (1.8)	23 (20.9)
Existence of texts regulating the vigilance system for the manufacturers of health products	50 (45.5)	9 (8.2)	51 (46.4)

### Participants’ opinions and experiences regarding ethical regulation of biomedical research

#### Degree of involvement in the preparation of research documents

Participants were distributed according to their experience or level of involvement in the development of some study documents such as study protocol and informed consent forms. About half of them have always or often been involved in such specific activities, while about the third have never done so (Table 3).

**Table 3:** Distribution of participants according to their involvement in the development of study protocol, briefing note and informed consent form (N = 110)

Study document	Always	Often	Sometimes	Never	No response
Study protocol	29 (26.4)	31 (28.2)	9 (8.2)	29 (26.4)	12 (10.9)
Briefing note	28 (25.5)	22 (20.0)	20 (18.2)	27 (24.5)	13 (11.8)
Informed consent form	28 (25.5)	26 (23.6)	18 (16.4)	27 (24.5)	11 (10.0)

#### Experience relating to the process of obtaining an ethics approval, providing information and obtaining informed consent

**Fig. 2:**
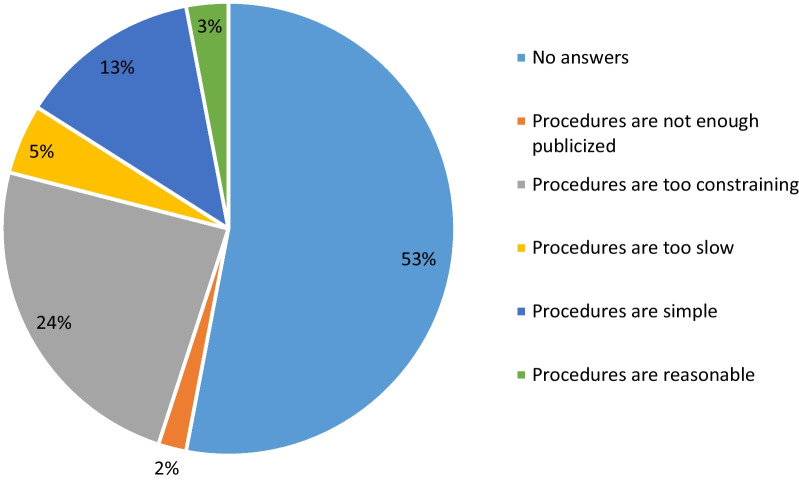
Distribution of participants according to their opinions on the CNERS procedures for protocol approval (N = 110)

About a quarter of the participants (24.0%) found very constraining the procedure of obtaining ethics approval while more than half did not answer the question (Fig. 2). About a quarter (25.5%) of the respondents declared they always present and properly explain the study briefing note to potential study participants before requiring their consent while 60.0% (n = 66) reported that their potential study participants are informed but to varying degrees. Finally, 20 (18.2%) of surveyed researchers acknowledged they never presented a briefing note to participants to obtain their consent while 13 abstained from answering the question. The reasons provided by 10 of the respondents to justify their failure to present the information note to their potential participants are summarised in Table 4 and were dominated by the perceived participant's inability to understand this information and the researchers’ difficulties to explain technical words to participants.

**Table 4:** Distribution of participants according to reasons motivating the lack of briefing note presentation before obtaining consent from study participants (N = 110)

Reasons	Number (%)		
	Yes	No	Unknown
Fear of high rate of participation refusal	2 (1.8)	8 (7.3)	100 (90.9)
Participants’ inability or difficulty to understand the briefing note	10 (9.1)	3 (2.7)	97 (88.2)
Difficulty to explain technical words in plain language for participants	10 (9.1)	3 (2.7)	97 (88.2)
Difficulty to translate the briefing note in local languages for illiterate participants	10 (9.1)	4 (3.6)	96 (87.3)
Others	5 (4.5)	3 (2.7)	102 (92.7)

#### Researchers’ opinions on the quality of the obtained consent

Six out of 10 researchers (62.7%) stated that they always obtain consent from participants during their studies. However, only 32.7% considered this consent to be really informed and this for several reasons: little opportunity to verify the level of understanding of what was the subject of consent; likely influence of a patriarchal society; intrinsic motivation of the study participant etc. Regarding the method of obtaining consent, 30.0% of participants declared obtaining it in writing for all of their studies, while 41.8% would obtain it in writing only in some of their studies. In 40% of cases, illiteracy was cited as the primary reason for choosing oral consent.

#### Withdrawal from studies

For some researchers, participants’ withdrawal from their studies was so common and would have happened always or very often according to 71 (68.2%) and 15 (3.6%) of them, respectively. Few of the respondents declared they have experienced it sometimes (7.0%; n = 7) or never (3.6%; n = 4). According to the researchers, the primary reasons evocated by withdrawing participants were personal convenience and time constraints.

## Discussion

The findings from this cross-sectional study on 110 bio-medical researchers affiliated to 13 research institutions from three main cities in the Southern region of Benin, revealed that they have limited knowledge regarding the CNERS and its functioning, the national ethical regulation of research and that their practice regarding ethics during their studies’ implementation, particularly the process of obtaining participants’ consent, is quite far from the optimal.

Regarding the knowledge of CNERS, it emerges from our study that CNERS was not well enough known to researchers, neither in its name, nor in its attributions, nor in the procedures for appointing its members. In fact, while all the respondents knew of the existence of the committee and its acronym (“CNERS”), only half of them could correctly define the latter. Regarding the attributions or functions, 25.5% provided the correct answers.

In his study conducted in Bamako (Mali) in 2010, Traoré reported that only 26.66% of the participants knew of the existence of the ethics committee and 13.33% knew of its functions [5]. However, Traore’s study involved, for 60%, people from the general population, outside the scientific community. In revenge, 20% of his sample was made of ethics committee’s members. Another study, conducted in Lebanon on knowledge and attitudes of physicians toward research ethics and scientific misconduct, reported that only 27.4% of participants were aware of the presence of the Lebanese National Consultative Committee on Ethics (LNCCE), with only half of them aware of its functions [6]. 

Our results revealed also, that basic ethics principles (Helsinki and Belmont report principles) were poorly known by the participants. This is in contrary with the findings from a study involving medical practitioners of a tertiary hospital in Nigeria and where the level of knowledge of these key ethics principles was rather higher with 85,7% of participants knowing the Helsinki principles and 90.5% those of the good clinical practices (GCP) [7]. But this high performance may be due to the study population which was entirely made of medical practitioners, as Helsinki and GCP principles are closely related to medical practices.

Regarding the knowledge of the national ethics code for biomedical research, few participants in our study were aware of such key items as the legal responsibility of researchers (34.5%), the concept of vulnerable person (33.6%), the legal criteria for the assessment of the benefits and risks (20%) and the conditions for including vulnerable people in research (25.5%). In the Lebanese study [6] only 25.7% of participants knew about the ethics charter and guiding principles of scientific research in Lebanon.

This kind of ignorance could be due to the fact that the publicity actions of the research governance system are insufficient or that despite the good intentions which guide it, ethics is not always welcomed by the scientific community and is seen as an obstacle to the science development [8]. Indeed, research ethics has attracted much criticism; it is time that universities, research associations and scientific institutions no longer content themselves with announcing that they are developing rigorous standards protecting the population against the potential risks of certain research projects, but that they promote places for interdisciplinary reflection on the very ethics of the researcher himself and his environment of practice [8].

About the third of the surveyed researchers acknowledged that they had never been involved in the development of research protocols or informed consent forms. This is likely due to the fact that most of the time, these tasks are primarily left to the principal investigator or research coordinator who develop these documents and share them with co-investigators for their comments. And some of the co-investigators, particularly those with poor knowledge in research ethics, may passively read the documents with no contributions.

In our study, about a quarter (25.5%) of the respondents declared they always present and properly explain the study briefing note to potential study participants before requiring their consent while 60.0% (n = 66) reported that their study participants are informed but to varying degrees. Finally, 20 (18.2%) of surveyed researchers acknowledged they never presented a briefing note to participants to obtain their consent while 13 abstained from answering the question. So, few of our study participants comply with the CIOMS requirements stipulated in its guidelines 9 and 10 [4]. In a Nigerian study, though all of the respondents agreed that obtaining informed consent was extremely important, only 52.4% of them declared obtaining it from study participants all the time, 42.9% some of the time, and 4.8% rarely [7]. As in our survey, illiteracy was reported as one of the major barriers to informed consent obtention. Indeed, illiteracy should not be an excuse for involving participants in studies without their informed consent, since researchers always manage to get their questionnaires understood by participants when seeking data from the latter. Furthermore, they should seek for the help of an impartial witness when needed. In a study conducted at a tertiary teaching medical college and hospital in Navi-Mumbai and involving postgraduate doctors, about 9% failed to explain informed consent to their participants in their local language, 5% neglected to hand over the participation sheet while obtaining informed consent and 19% neglected to seek for the signature of an impartial witness when required to do so [9].

### Limitations and strengths of the study

The primary weakness of the present study is the possibility of selection bias as only half of the approached potential participants were eligible and included in the analysis. As the objectives of the study were related to knowledge, opinion and practice or behaviours, it may be assumed that those who declined their participation to the study or abstained from responding might primarily be those with poor knowledge or bad practices or behaviours. From this perspective, the prevalence of poor knowledge or improper practices in relation to research ethics, might have been under-estimated. Moreover, a social desirability bias is possible as well and could have caused a further under-estimation of bad practices. However, the anonymous data collection and the online administration of the questionnaire for some participants might have reduced this bias.

Assessment and recall biases were possible but limited by defining clear items with standardized responses, or using open or multiple-choice questions, when possible. This was made possible by pre-testing and validating the questionnaire before its use. Finally, the sample size was relatively small, (110 participants compared to our calculated minimal sample size of 134), which could have affected the precision of the results. Indeed, using our actual sample size of 110 and the primary finding of 25% for the proportion of participants knowing CNERS’s attributions or functions, we found a calculated power of 0.70, which is lower than the typical 0.80.

The main strength of this study is that it was the first one in Benin to address the issue of researchers’ knowledge and opinions regarding the national regulatory mechanism of research. Regarding the study design, we carried out a descriptive cross-sectional study which is suitable to answer the research questions.

In sum, this research reveals the need to ensure better visibility of the national ethics committee for health research (CNERS) of Benin, through the development of an active partnership with research institutions with the creation of a reflection framework gathering researchers and CNERS members, training of the various research actors including researchers, training of members of ethics committees at regular intervals; popularization of its procedures; advocacy to set up more institutional ethics committees; the creation of a national directory of health research. To enable CNERS to handle a larger volume of studies, to carry out on-site review and follow-up in addition to protocol review, it would need a well-functioning secretariat, properly trained members and substantial funding [10].

## Conclusions

In conclusion, the foundations of a good ethical framework for health research exist in Benin. However, the deployment and use of the various legal texts deserve to be improved.

This study opens up other research perspectives, including the evaluation of the level of compliance of researchers with the research ethics and deontology code in Benin, with the ethics principles in clinical trials and with procedures for the storage and preservation of biological materials for the purposes of subsequent research.

## Data Availability

The datasets used and/or analysed during the current study are not publicly available because this is forbidden by our research ethics committee, unless we ask for a permission to do so. However, these datasets are available from the corresponding author on reasonable request.

## Abbreviations

CNERS: Comité National d’Ethique pour la Recherche en Santé
CIOMS: Council for International Organizations of Medical Sciences
REC: Research Ethics Committee
IRB: Institutional Review Board
CHUMEL: Centre Hospitalier Universitaire de la Mère et de l’Enfant Lagune
CHUD-OP: Centre Hospitalier Universitaire Départemental de Ouémé-Plateau
CREC: Centre de Recherche Entomologique de Cotonou
CERRHUD: Centre de Recherche en Reproduction Humaine et en Démographie
PNLP: Programme National de Lutte contre le Paludisme
ISBA: Institut des Sciences Biomédicales Appliquées
IRD: Institut de Recherche et Développement
ABMS: Association Béninoise de Marketing Social
PSI: Population Services International
